# 

**Published:** 1903-07

**Authors:** 


### INDEX TO VOLUME XIX.

Appendicitis . . . . . . . . . . . . . . . . . . . . . . . . . . . . . . . . . . . . . . . . . 1, 260
A Symposium; Otitis Media . . . . . . . . . . . . . . . . . . . . . . . . . . 9
Austin District Medical Society. . . . . . . . . . . . . . . . . . . . . . . . . 19
American Congress on Tuberculosis . . . . . . . . . . . . . . . . . . . . . 72
American Medical Association; Reorganization. . . . . . . . . . . . 84
Animal Extracts, Therapeutic Value of. . . . . . . . . . . . . . . . . . 83
Adrenalin Chloride in Dentistry. . . . . . . . . . . . . . . . . . . . . . . . 115
Anesthol, Report on Use of. . . . . . . . . . . . . . . . . . . . . . . . . . . 197
Abscess of the Liver, Tropical. Thompson . . . . . . . . . . . . . . . 335
Auto-toxemia . . . . . . . . . . . . . . . . . . . . . . . . . . . . . . . . . . . . . 500

Contagious Diseases and Country Newspapers. . . . . . . . . . . . . 161
Congress on Tuberculosis, The International, and Its Critics,
St. Louis, October, 1904. . . . . . . . . . . . . . . . . . . 312, 326, 403, 409
Convention of Texas Health Officers. . . . . . . . . . . . . . . . . . . . 379

Doctors and "Doctors" . . . . . . . . . . . . . . . . . . . . . . . . . . . . . . 169
Diagnosis, Pathology and Treatment. Leake. . . . . . . . . . . . . . 443

Experience vs. Theory . . . . . . . . . . . . . . . . . . . . . . . . . . . . . . 7
Experiments with Morris Formaldehyde Generator. . . . . . . . . 41

Finsen Light Cure, The. . . . . . . . . . . . . . . . . . . . . . . . . . . . . . 302
Formaldehyde Generator. Morris . . . . . . . . . . . . . . . . . . . . . . 41

Hadra, Dr. B. E., Death of. . . . . . . . . . . . . . . . . . . . . . . . . . . . 11
Higher Education, The Effects of on Women. . . . . . . . . . . . . . 155

Intestinal Obstruction by Merkel's Diverticulum. . . . . . . . . . . 305

Lying-in Chamber, Preparation of. . . . . . . . . . . . . . . . . . . . . . 59

Murphy, Dr. J. B. . . . . . . . . . . . . . . . . . . . . . . . . . . . . . . . . . 79

Nephritis in Summer . . . . . . . . . . . . . . . . . . . . . . . . . . . . . . . [illegible]

Prostatectomy, Indications and Technique . . . . . . . . . . . . . . . 117
Physician and Homeopath; Their Relation in Practice. . .89, 164

Railway Sanitation, Conference on . . . . . . . . . . . . . . . . . . . . . [illegible]6

Rape, The Cause and Prevention of. Daniel. [illegible] . . . . . . . . . [illegible]

Respectfully Submitted . . . . . [illegible] . . . . . . [illegible]

Report of a Case of Intractable Vom[illegible]

Treated by Electric Currents and Illust[illegible]ng the In[illegible]

tance of Current Differentiation . . . . . . . . . . . . . . . . . . . . [illegible]

431960

State Medical Association of Texas, Thirty-sixth Annual Meeting . . . . . . . . . . . . . . . . . . . . . . . . . . . . . . . . . . . . . . . . 353
State Medical Association of Texas. . . . . . . . . . . . . . . . . . .112, 392
State Medical Association, April, 1904, Meeting. . . . . . . . . . . 464
Stegomyia Fasciata . . . . . . . . . . . . . . . . . . . . . . . . . . . . . . .418, 462
Stout, Dr. S. H., Death of. . . . . . . . . . . . . . . . . . . . . . . . . . . . 128
Sanitary Law, The New Texas. . . . . . . . . . . . . . . . . . . . . . . . . 270
Sadism in the Negro . . . . . . . . . . . . . . . . . . . . . . . . . . . . . . . . 452
Snakebite, A Case of; Adrenalin in. . . . . . . . . . . . . . . . . . . . . . 87
Situation, The (Yellow Fever at San Antonio) . . . . . . . . . . . 167

Talipes; Tenotomy in . . . . . . . . . . . . . . . . . . . . . . . . . . . . . . . 55
The Great White Plague . . . . . . . . . . . . . . . . . . . . . . . . . . . . . 63
The Relation of Public Schools to the Medical Profession. . . 495
Tonsilitis . . . . . . . . . . . . . . . . . . . . . . . . . . . . . . . . . . . . . . . . 301

Wyeth, Dr. Jno. A., Letter from. . . . . . . . . . . . . . . . . . . . . . . 417
West, D. H. A. In Memoriam. . . . . . . . . . . . . . . . . . . . . . .358, 268

Yellow Fever . . . . . . . . . . . . . . . . . . . . . . . . . . . . . . . . . . . . . . 92
Yellow Fever Epidemic at Columbus, 1873. . . . . . . . . . . . . . . 164
Yellow Fever. Does the Germ of Have Home in Mosquito?. 291
Yellow Fever in San Antonio, 1903. . . . . . . . . . . . . . . . . . .208-228
Yellow Fever, the Influence of Environment in. . . . . . . . . . . . 247
Yellow Fever, Differential Diagnosis in. . . . . . . . . . . . . . . . . 255

